# In medical-surgical ICU patients, major bleeding is common but independent of heparin prophylaxis

**DOI:** 10.1186/cc11042

**Published:** 2012-03-20

**Authors:** F Lauzier, D Arnold, C Rabbat, D Heels-Ansdell, P Dodek, B Ashley, M Albert, K Khwaja, M Ostermann, Y Skrobik, R Fowler, L McIntyre, J Nates, T Karachi, R Lopes, N Zytaruk, M Crowther, D Cook

**Affiliations:** 1 CHA-Hôpital de l'Enfant-Jésus, Université Laval, Québec, Canada; 2 McMaster University, Hamilton, Canada; 3 University of British Columbia, Vancouver, Canada; 4 Université de Montréal, Canada; 5 Université McGill, Montréal, Canada; 6 King's College London, UK; 7 University of Toronto, Canada; 8 Université d'Ottawa, Canada; 9 MD Anderson Hospital, Houston, TX, USA; 10 Duke Clinical Research Institute, Durham, NC, USA

## Introduction

Bleeding frequently complicates critical illness. Our objectives were to describe the incidence, locations and predictors of major bleeding in patients with low risk of bleeding receiving thromboprophylaxis.

## Methods

In the PROTECT trial comparing dalteparin to unfractionated heparin for thromboprophylaxis in medical-surgical ICU patients, research coordinators used a validated ICU-specific tool to describe the site and severity of each bleeding event, which was reevaluated by two independent blinded adjudicators. Patients with trauma, orthopedic surgery or neurosurgery were excluded. Major bleeding was defined as life threatening, occurring in critical sites, requiring ≥2 units of red blood cells or an invasive intervention, or associated with an unexplained decrease in systolic blood pressure (≥20 mmHg) or increase in heart rate (≥20 beats/minute). We used Cox proportional hazard models adjusting for age, APACHE II, reason for ICU admission, end-stage renal disease, drugs affecting coagulation, coagulation parameters and life-support interventions to identify predictors of bleeding.

## Results

Among 3,746 patients, 208 had major bleeding (5.6%, 95% CI 4.9 to 6.3%). The commonest bleeding sites were: gastrointestinal tract (51.9%), surgical site (30.3%), respiratory tract (15.9%), retroperitoneal (8.2%) and intracranial (3.4%). Independent predictors of major bleeding (expressed as hazard ratio with 95% CI) were: prolonged activated partial thromboplastin time (aPTT) (1.10, 1.05 to 1.14 per 10-second increase), thrombocytopenia (1.16, 1.09 to 1.24 per 50 × 10^9^/l decrease in platelet count), therapeutic heparin (3.26, 1.72 to 6.17), anti-platelet agents (that is, acetylsalicylic acid and/or clopidogrel) (1.38, 1.02 to 1.88), renal replacement therapy (1.75, 1.20 to 2.56) and surgery in the preceding 3 days (1.64, 1.01 to 2.65). Prophylactic dalteparin in the preceding 3 days was not associated with bleeding.

## Conclusion

Major bleeding occurred in 5.6% of medical-surgical ICU patients. Prolonged aPTT, thrombocytopenia, therapeutic (but not prophylactic) heparin, anti-platelet agents and recent surgery are potentially modifiable and independent predictors of bleeding.

